# Prognostic Value of the National Early Warning Score Combined with Nutritional and Endothelial Stress Indices for Mortality Prediction in Critically Ill Patients with Pneumonia

**DOI:** 10.3390/medicina62010207

**Published:** 2026-01-19

**Authors:** Ferhan Demirer Aydemir, Murat Daş, Özge Kurtkulağı, Ece Ünal Çetin, Feyza Mutlay, Yavuz Beyazıt

**Affiliations:** 1 Department of Internal Medicine, Division of Intensive Care Medicine, Faculty of Medicine, Çanakkale Onsekiz Mart University, Çanakkale 17020, Türkiye; 2 Department of Emergency Medicine, Faculty of Medicine, Çanakkale Onsekiz Mart University, Çanakkale 17020, Türkiye; muratdas58@gmail.com; 3 Department of General Internal Medicine, Faculty of Medicine, Çanakkale Onsekiz Mart University, Çanakkale 17020, Türkiye; ozgekurtkulagi@gmail.com (Ö.K.); eceunalcetin@gmail.com (E.Ü.Ç.); 4 Department of Internal Medicine, Division of Geriatrics, Faculty of Medicine, Çanakkale Onsekiz Mart University, Çanakkale 17020, Türkiye; feyzamutlay@gmail.com; 5 Department of Internal Medicine, Division of Gastroenterology, Faculty of Medicine, Çanakkale Onsekiz Mart University, Çanakkale 17020, Türkiye; yavuzbeyaz@yahoo.com

**Keywords:** pneumonia, intensive care unit, mortality, prognosis, early warning score, biomarkers

## Abstract

*Background and Objectives*: Pneumonia is a leading cause of intensive care unit (ICU) admission and is associated with high mortality, particularly among patients with multiple comorbidities. Accurate early risk stratification is essential for guiding clinical decision-making in critically ill patients. However, the prognostic benefit of combining clinical scoring systems with nutritional and endothelial stress indices in ICU patients with pneumonia remains unclear. *Materials and Methods*: This retrospective, single-center cohort study included adult patients admitted to the ICU with a diagnosis of pneumonia between 1 January 2023 and 1 July 2025. Demographic characteristics, comorbidities, clinical variables, laboratory parameters, and prognostic scores were obtained from electronic medical records. The National Early Warning Score (NEWS), Prognostic Nutritional Index (PNI), and Endothelial Activation and Stress Index (EASIX) were calculated at ICU admission. The primary outcome was in-hospital mortality. Univariate and multivariate logistic regression analyses were performed to examine variables associated with in-hospital mortality. The discriminative performance of individual and combined prognostic models was evaluated using receiver operating characteristic (ROC) curve analysis. *Results*: A total of 221 patients were included; 79 (35.7%) survived and 142 (64.3%) died during hospitalization. Non-survivors had significantly higher NEWS and EASIX values and lower PNI values compared with survivors (all *p* < 0.05). In multivariate analysis, endotracheal intubation (OR: 12.46; *p* < 0.001), inotropic use (OR: 5.14; *p* = 0.001), and serum lactate levels (OR: 1.75; *p* = 0.003) were identified as being independently associated with in-hospital mortality. Models combining NEWS with PNI or EASIX demonstrated improved discriminatory performance. *Conclusions*: In critically ill patients with pneumonia, integrating NEWS with nutritional and endothelial stress indices provides numerically improved discrimination compared with NEWS alone, although the incremental gain did not reach statistical significance.

## 1. Introduction

Pneumonia remains one of the leading causes of admission to the intensive care unit (ICU) and is associated with substantial short-term mortality, particularly in patients with significant comorbidity and severe physiological derangement [1]. Despite advances in antimicrobial therapy and supportive care, outcomes in critically ill patients with pneumonia remain poor, underscoring the need for accurate early risk stratification to guide clinical decision-making and resource allocation in the ICU setting [2].

Early warning scores have been widely adopted to identify clinical deterioration and estimate mortality risk in hospitalized patients with infection [3]. Among these, the National Early Warning Score (NEWS) and its updated version, NEWS2, have demonstrated acceptable performance in predicting adverse outcomes in patients with pneumonia across different clinical settings [4]. However, previous studies indicate that although NEWS effectively reflects acute physiological instability, its prognostic accuracy may be limited when used in isolation, particularly in critically ill patients with complex systemic involvement [5,6].

Beyond physiological instability, host-related factors such as nutritional status and immune competence play a crucial role in determining outcomes in severe infections [7]. Malnutrition is common among critically ill patients with pneumonia and has been linked to impaired immune response, delayed recovery, and increased mortality [8]. The Prognostic Nutritional Index (PNI), derived from the serum albumin and lymphocyte count, has emerged as a practical marker reflecting both nutritional reserve and immune function [9]. Recent studies have demonstrated that lower PNI values are associated with increased mortality in patients with pneumonia and other critical illnesses [10,11].

In parallel, endothelial dysfunction has increasingly been recognized as a central pathophysiological mechanism in severe pneumonia and sepsis [12]. Endothelial injury contributes to microvascular dysfunction, coagulopathy, and impaired tissue perfusion, ultimately leading to multi-organ failure [13]. The Endothelial Activation and Stress Index (EASIX), calculated using routinely available laboratory parameters including lactate dehydrogenase, serum creatinine, and platelet count, has been shown to predict short- and intermediate-term mortality in critically ill and septic ICU populations [14]. However, data specifically addressing the prognostic role of EASIX in ICU patients with pneumonia remain limited.

Serum lactate is a well-established biomarker of disease severity in critical illness, reflecting global tissue hypoperfusion and circulatory failure [15]. Elevated lactate levels at ICU admission have consistently been associated with increased mortality in patients with pneumonia, as supported by recent cohort studies and meta-analyses [16,17]. Taken together, these findings suggest that prognostic assessment in ICU pneumonia may benefit from a multidimensional approach integrating acute physiological derangement, host nutritional–immune status, and endothelial stress.

Accordingly, the present study aimed to evaluate the prognostic value of combining NEWS with the Prognostic Nutritional Index and the Endothelial Activation and Stress Index for predicting in-hospital mortality in critically ill patients with pneumonia. We hypothesized that integrating these complementary domains would improve early risk stratification beyond single-score approaches and provide a practical, bedside-applicable tool for use in the ICU.

## 2. Materials and Methods

### 2.1. Study Design and Setting

This study was designed as a retrospective, single-center cohort study conducted in the Intensive Care Unit (ICU) of the Department of Internal Medicine, Çanakkale Onsekiz Mart University Faculty of Medicine. Adult patients admitted to the ICU with a diagnosis of pneumonia between 1 January 2023 and 1 July 2025 were retrospectively evaluated using electronic medical records.

### 2.2. Study Population

Pneumonia was diagnosed based on clinical presentation, laboratory findings, and/or radiological evidence, in accordance with standard clinical practice. Patients were identified using the primary ICU admission diagnosis field for pneumonia in the electronic hospital system, and eligibility was confirmed by admission notes, chest imaging, and initiation of pneumonia-directed antimicrobial therapy.

Patients were eligible for inclusion if they met all of the following criteria: (1) age ≥ 18 years, (2) admission to the ICU with a primary diagnosis of pneumonia, and (3) availability of complete clinical, laboratory, and scoring data in the hospital information system.

Patients were excluded if they had missing key clinical or laboratory data, were discharged or transferred to another center within the first 24 h of ICU admission, or were admitted to the ICU for reasons other than pneumonia. Due to the retrospective design of the study and the limitations of electronic medical records, a reliable and standardized classification of pneumonia phenotypes (community-acquired, hospital-acquired, ventilator-associated, or aspiration pneumonia) was not consistently available for all patients. Therefore, pneumonia phenotype-specific subgroup analyses were not performed. All patients were managed in a single tertiary-care intensive care unit using standardized diagnostic and treatment protocols for pneumonia.

### 2.3. Data Collection

Data were extracted retrospectively from the hospital electronic database and patient medical records.

Demographic and clinical variables included age, sex, nursing home residency, and comorbidity, which was defined as the presence of at least one chronic medical condition at ICU admission. Comorbid conditions included diabetes mellitus, chronic obstructive pulmonary disease, chronic kidney disease, chronic liver disease, heart failure, coronary artery disease, cerebrovascular disease, malignancy, immunosuppression, and psychiatric disorders. Clinical course-related variables included the requirement for endotracheal intubation, use of inotropic agents, and length of ICU stay. Endotracheal intubation and inotropic support were recorded if they occurred at any time during the ICU stay and were not restricted to admission status. Severity scores such as APACHE II, SOFA, or the Vasoactive–Inotropic Score (VIS) were not routinely recorded in the electronic medical records during the study period and therefore could not be included in the analysis.

All key candidate predictors, including NEWS components, serum albumin, lymphocyte count, lactate dehydrogenase, creatinine, platelet count, serum lactate, procalcitonin, and C-reactive protein, are routinely measured as part of standard care for patients admitted to the intensive care unit. The original National Early Warning Score (NEWS) was used in this study. NEWS2 was not applied, as oxygen supplementation data required for NEWS2 were not consistently available in this retrospective dataset. Accordingly, there were no missing data for variables required for the calculation of NEWS, PNI, or EASIX in the final analytical cohort. Patients were excluded during the screening phase based on predefined clinical and administrative exclusion criteria, including non-pneumonia ICU admission, early transfer/discharge, and duplicate ICU admissions. These exclusion criteria and the corresponding number of excluded patients are detailed in Supplementary Table S1. All clinical and laboratory data were retrieved from the hospital electronic medical record and laboratory information system (MIA Hospital Information Management System, MIA-MED Information Technologies, Ankara, Turkey).

### 2.4. Clinical Scores and Indices

The National Early Warning Score (NEWS) was calculated using vital signs recorded at ICU admission. The Prognostic Nutritional Index (PNI) was calculated using the following formula, PNI = [10 × serum albumin (g/dL)] + [0.005 × total lymphocyte count (per mm^3^)], as previously described. The Endothelial Activation and Stress Index (EASIX) was calculated as (lactate dehydrogenase [U/L] × serum creatinine [mg/dL])/platelet count (10^9^/L). For all indices and laboratory parameters, the first available measurement obtained within the first 24 h of ICU admission was used for analysis. NEWS was calculated from vital signs recorded at ICU admission, whereas PNI and EASIX were derived from the first laboratory measurements obtained within the first 24 h after ICU admission. All laboratory parameters were recorded as the first measured values obtained within the first 24 h following ICU admission.

### 2.5. Laboratory Parameters

Laboratory parameters obtained at ICU admission included arterial pH, serum lactate, urea, white blood cell count, albumin, C-reactive protein (CRP), procalcitonin, ferritin, and D-dimer levels.

### 2.6. Outcome Measure

The primary outcome of the study was in-hospital mortality. Patients were categorized into two groups according to survival status: survivors and non-survivors.

### 2.7. Statistical Analysis

All statistical analyses were performed using SPSS version 25.0 (IBM Corp., Armonk, NY, USA). The normality of continuous variables was assessed using the Shapiro–Wilk test. Normally distributed variables were expressed as mean ± standard deviation, while non-normally distributed variables were presented as medians (interquartile range). Categorical variables were reported as counts and percentages.

Continuous variables were compared using Student’s *t*-test or Mann–Whitney U test, as appropriate. Categorical variables were compared using the chi-square test or Fisher’s exact test.

Univariate logistic regression analyses were performed to identify variables associated with in-hospital mortality. Variables with a *p*-value < 0.10 in univariate analysis were entered into multivariate logistic regression models, and results were reported as odds ratios (ORs) with 95% confidence intervals (CIs). Model calibration for the multivariate logistic regression was assessed using the Hosmer–Lemeshow goodness-of-fit test.

The discriminative performance of NEWS, PNI, and EASIX, both individually and in combination, was evaluated using receiver operating characteristic (ROC) curve analysis. The area under the ROC curve (AUC) was calculated to assess predictive accuracy, and comparisons between AUCs were performed using the DeLong test. A two-sided *p*-value < 0.05 was considered statistically significant. For the interaction analysis, optimal cutoffs for NEWS, PNI, and EASIX were determined using the Youden Index to maximize predictive performance. The multivariate models were adjusted for age, sex, and comorbidity.

No artificial intelligence (AI)–based tools or algorithms were used for data extraction, analysis, outcome assessment, or decision-making in this study.

### 2.8. Ethical Considerations

This study was conducted in accordance with the principles of the Declaration of Helsinki and was approved by the Non-Interventional Clinical Research Ethics Committee of Çanakkale Onsekiz Mart University (approval number: 2025-318; meeting date: 8 October 2025). Given the retrospective nature of the study and the use of anonymized data, the requirement for informed consent was waived.

## 3. Results

### 3.1. Patient Characteristics

A total of 221 patients admitted to the ICU with a diagnosis of pneumonia were included in the study. Among them, 79 patients (35.7%) survived, while 142 patients (64.3%) died during hospitalization. The mean age of the study population was 74.8 ± 14.1 years, with no significant difference between survivors and non-survivors (*p* = 0.193). Sex distribution was similar between groups (male sex: 49.4% vs. 56.3%, *p* = 0.319), and nursing home residency did not differ significantly (*p* = 0.514). The presence of comorbidities was significantly more frequent among non-survivors (96.5% vs. 89.9%, *p* = 0.047). A history of stroke was more common among survivors compared with non-survivors (22.5% vs. 10.1%, *p* = 0.022), whereas other comorbid conditions showed no statistically significant differences. Regarding prognostic indices, PNI values were significantly lower in non-survivors [31.5 (28.4–37.4) vs. 35.3 (31.5–39.6), *p* = 0.001], while EASIX values were significantly higher [2.6 (1.2–6.8) vs. 2.0 (0.8–3.6), *p* = 0.021]. NEWSs were also significantly higher in non-survivors compared with survivors (9.6 ± 2.9 vs. 7.8 ± 3.1, *p* < 0.001). Endotracheal intubation and inotropic support were required significantly more often in non-survivors (82.4% vs. 24.1% and 59.2% vs. 17.7%, respectively; both *p* < 0.001) (Table 1).

### 3.2. Laboratory Findings

Non-survivors had significantly lower arterial pH values and higher serum lactate levels compared with survivors [2.3 (1.5–3.7) vs. 1.4 (1.0–1.9) mmol/L, *p* < 0.001]. Serum albumin levels were also significantly reduced in non-survivors [2.8 (2.4–3.1) vs. 3.1 (2.8–3.4) g/dL, *p* < 0.001]. In addition, procalcitonin and ferritin levels were significantly higher in patients who died (*p* < 0.001 and *p* = 0.007, respectively) (Table 1).

### 3.3. Logistic Regression Analysis

In the univariate logistic regression analysis, PNI, EASIX, NEWS, ICU length of stay, endotracheal intubation, inotropic use, serum lactate, albumin, procalcitonin, and ferritin were significantly associated with in-hospital mortality. In the multivariate logistic regression model, endotracheal intubation (OR: 12.46; 95% CI: 4.66–33.30; *p* < 0.001), inotropic use (OR: 5.14; 95% CI: 1.99–13.23; *p* = 0.001), and serum lactate level (OR: 1.75; 95% CI: 1.21–2.51; *p* = 0.003) showed the strongest statistical associations with in-hospital mortality. Although NEWS, EASIX, and PNI were included in the multivariable model, they did not retain independent significance after adjustment for clinical and laboratory covariates. The multivariate model demonstrated adequate calibration, with no significant difference between predicted and observed mortality risks (Hosmer–Lemeshow test: *X*^2^ = 10.329, df = 8, *p* = 0.243) (Table 2). Calibration assessment in this study was limited to the Hosmer–Lemeshow test, and no calibration slope, intercept, or calibration plot was generated.

### 3.4. Interaction Analysis of NEWS with PNI and EASIX

Patients with NEWS > 7 and PNI < 34.7 exhibited the highest mortality rate (78.6%), corresponding to a 6.45-fold increased risk compared with the reference group (*p* < 0.001). Similarly, patients with NEWS > 7 and EASIX ≥ 3.3 demonstrated a mortality rate of 79.4%, with an adjusted odds ratio of 7.37 (*p* < 0.001) (Table 3). Because these cutoffs were derived in a data-driven manner from the same cohort, they should be considered exploratory and require external validation before being used as clinical decision thresholds.

### 3.5. Discriminative Performance and ROC Curve Analysis

**Figure 1:**
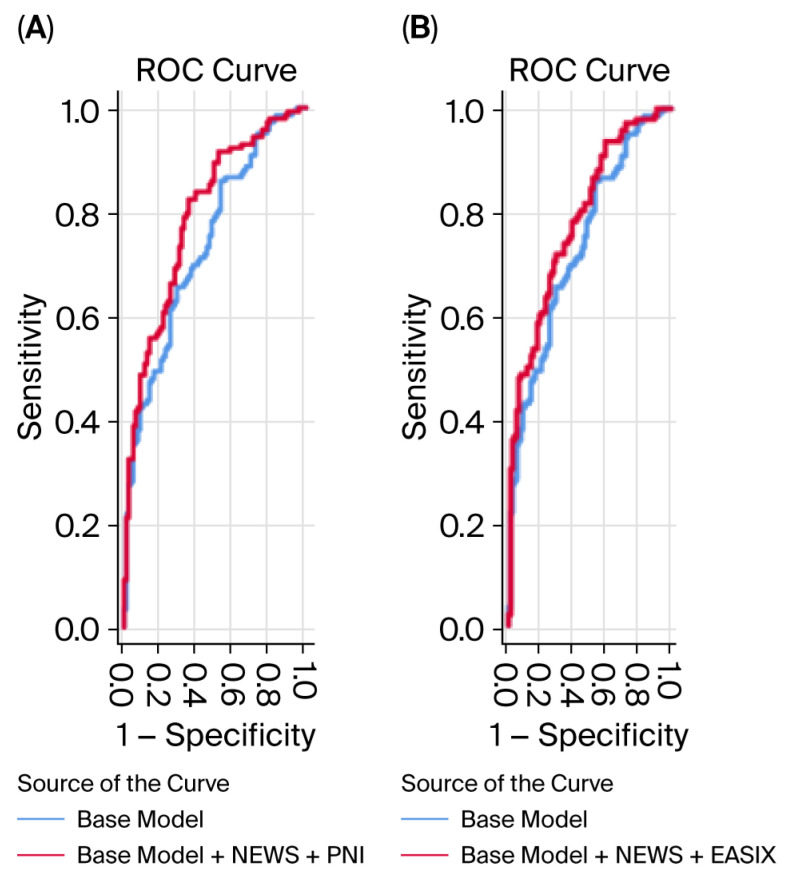
Receiver operating characteristic (ROC) curves for predicting in-hospital mortality in critically ill patients with pneumonia. (**A**) ROC curves comparing the discriminative performance of the base model (age, sex, serum lactate, procalcitonin, and C-reactive protein) and the base model combined with the National Early Warning Score (NEWS) and the Prognostic Nutritional Index (PNI). The addition of NEWS and PNI resulted in a significant improvement in predictive accuracy compared with the base model alone. (**B**) ROC curves comparing the base model with the base model combined with the National Early Warning Score (NEWS) and the Endothelial Activation and Stress Index (EASIX). Incorporation of NEWS and EASIX significantly enhanced the discriminative ability for in-hospital mortality prediction. Abbreviations: ROC, receiver operating characteristic; NEWS, National Early Warning Score; PNI, Prognostic Nutritional Index; EASIX, Endothelial Activation and Stress Index.

ROC curve analysis demonstrated that NEWS, PNI, and EASIX individually showed moderate discriminative ability for predicting in-hospital mortality. The addition of PNI or EASIX to NEWS-based models significantly improved predictive performance. The base model combined with NEWS and PNI yielded an AUC of 0.791 (95% CI: 0.731–0.852), while the base model combined with NEWS and EASIX achieved an AUC of 0.785 (95% CI: 0.724–0.845). Pairwise comparisons using the DeLong test confirmed that both combined models provided a statistically significant incremental discriminative value over the base model alone (*p* = 0.025 and *p* = 0.027, respectively). However, when compared specifically to the model already containing NEWS, the additional incremental value did not reach statistical significance (*p* = 0.099 and *p* = 0.068), indicating that while the combined models are strong, the standalone statistical contribution of PNI/EASIX over NEWS is modest (Table 4 and Table 5, Figure 1).

## 4. Discussion

In this retrospective cohort of critically ill adults admitted to the intensive care unit with pneumonia, our results indicate that mortality risk is not sufficiently captured by physiological severity alone. When bedside early warning scores were interpreted together with indices reflecting nutritional–immune reserve and endothelial stress, prognostic performance improved in a clinically meaningful way, highlighting the value of a multidimensional approach to early risk stratification. Patients who did not survive had higher admission NEWS and EASIX values and lower PNI levels, and combining NEWS with either PNI or EASIX resulted in better discrimination than reliance on a single score. Alongside these composite measures, endotracheal intubation, inotropic support, and elevated admission serum lactate remained independently associated with in-hospital mortality, emphasizing the impact of advanced organ failure and shock physiology in this setting. These variables reflect downstream disease severity and treatment escalation rather than baseline risk at ICU admission and should therefore be interpreted as clinical course markers rather than prognostic factors present at admission.

NEWS and NEWS2 are widely used to identify early clinical deterioration and estimate mortality risk in pneumonia [4,18]. Prior work has consistently linked higher NEWS values to short-term mortality, particularly in older populations, although reported accuracy varies across disease severity and care settings [19]. In our ICU cohort, admission NEWS was clearly higher among non-survivors, supporting its continued relevance even in advanced disease. At the same time, the present findings suggest that physiological scoring alone may not fully reflect the underlying vulnerability of critically ill patients. By incorporating markers of systemic stress and host reserve, our findings extend previous observations and suggest that risk estimation in ICU pneumonia can be refined beyond vital sign-based scores alone.

The role of nutritional status and immune competence in determining outcomes from severe infection has gained increasing attention [20,21]. The Prognostic Nutritional Index, based on serum albumin and lymphocyte count, has previously been associated with mortality in pneumonia cohorts [10,22]. We observed a similar pattern, with significantly lower PNI values among non-survivors. From a clinical perspective, this likely reflects diminished physiological reserve and impaired immune response, both of which may amplify susceptibility to adverse outcomes in ICU pneumonia. Importantly, the addition of PNI to the NEWS model resulted in a numerical increase in discriminative performance (AUC 0.791 vs. 0.763). Although this incremental difference did not reach statistical significance (*p* = 0.099) in pairwise comparison, the observed trend suggests that integrating host-related vulnerability with acute physiological derangement may provide added prognostic insight in critically ill pneumonia populations.

Endothelial dysfunction represents another pathophysiological dimension that may be underappreciated in routine bedside assessment [23]. EASIX has been shown to predict mortality in septic ICU patients and other critically ill populations [24,25,26]. Although pneumonia-specific data remain limited, the association between higher EASIX values and mortality in our cohort is biologically plausible. Widespread endothelial injury, microvascular dysfunction, and coagulation abnormalities are well-recognized features of severe pneumonia-related organ failure [12]. Our findings extend existing evidence by demonstrating that endothelial stress indices convey complementary prognostic information when interpreted alongside NEWS in an ICU pneumonia cohort, rather than simply duplicating physiological severity. The improved performance of the NEWS + EASIX model supports this concept.

Serum lactate showed a strong association with in-hospital mortality in our analysis. This observation is consistent with prior studies and meta-analyses demonstrating that elevated lactate levels reflect tissue hypoperfusion and are associated with worse outcomes in pneumonia [27,28]. In practice, lactate remains one of the most accessible indicators of circulatory failure, and its prognostic signal in our cohort reinforces its central role in ICU risk stratification. Similarly, the independent associations of endotracheal intubation and inotropic use with mortality are clinically intuitive. Both variables signal advanced respiratory and hemodynamic compromise and have repeatedly been linked to poor outcomes in severe pneumonia [29]. It should be noted, however, that these factors also reflect treatment decisions, which may partly confound their relationship with outcome.

In recent years, machine learning-based models have reported impressive discrimination for predicting mortality in ICU patients with pneumonia [30,31]. Despite their accuracy, these approaches often depend on complex computational infrastructure and limited transparency, which may restrict bedside adoption. In contrast, the novelty of the present approach lies in demonstrating that routinely available, easily interpretable clinical parameters can be meaningfully combined to improve prognostic performance without sacrificing practicality. The combined models examined in this study rely exclusively on routinely available clinical and laboratory parameters, are simple to calculate, and do not require specialized tools. The observed AUC values for NEWS + PNI (0.791) and NEWS + EASIX (0.785) suggest that clinically pragmatic models can still achieve meaningful prognostic performance.

Overall, these findings support a multidimensional approach to risk stratification in critically ill patients with pneumonia. Rather than proposing a new prognostic score, our results highlight the added value of integrating complementary biological domains—physiological instability, nutritional–immune reserve, and endothelial stress—within a single, bedside-applicable framework. Integrating NEWS with indices of nutritional–immune status and endothelial stress appears to improve mortality prediction and may allow earlier identification of patients at particularly high risk, thereby supporting more individualized ICU management strategies.

### 4.1. Clinical Implications

From a practical standpoint, the combined use of NEWS with PNI or EASIX enables rapid risk assessment at the time of ICU admission using data that are already part of routine care. Patients presenting with high NEWS values together with low PNI or elevated EASIX seem to represent a subgroup with particularly unfavorable prognosis. The clinical novelty lies in identifying this high-risk phenotype using parameters that are already available at admission, without the need for additional testing or complex modeling. Recognizing this pattern early may encourage closer monitoring, earlier anticipation of respiratory or hemodynamic deterioration, and timely involvement of multidisciplinary teams.

These combined indices may also help guide more efficient use of ICU resources, especially in settings where capacity is limited. Identifying patients at increased risk of in-hospital mortality could support tailored interventions, such as early nutritional optimization, intensified hemodynamic surveillance, and prompt escalation of care when indicated. In addition, the use of transparent and easily interpretable prognostic tools may facilitate more informed discussions with patients’ families regarding prognosis and goals of care.

### 4.2. Strengths and Limitations

This study has several strengths. It evaluates complementary prognostic domains—physiological instability, nutritional–immune status, and endothelial stress—using variables that are readily available in everyday ICU practice. By examining their combined rather than isolated prognostic value in an ICU pneumonia cohort, this study adds incremental evidence to an area where data remain limited. The use of multivariable regression and ROC analyses allowed us to determine whether combining these measures offered incremental prognostic value beyond individual scores.

Several limitations should be acknowledged. The retrospective, single-center design limits causal inference and may affect generalizability. Established ICU severity scores such as SOFA or APACHE II were not available in this dataset, which restricts adjustment for baseline disease severity and raises the possibility of residual confounding. Variables such as endotracheal intubation and inotropic support likely reflect advanced disease severity and therapeutic decision-making rather than baseline prognostic factors; therefore, causal interpretations should be avoided. Although key clinical variables were included, unmeasured factors such as pneumonia etiology, timing of antimicrobial therapy, ventilatory strategies, or fluid management cannot be excluded as contributors to outcome. In addition, only admission values were analyzed, and dynamic changes in NEWS, PNI, EASIX, or lactate during the ICU stay may provide additional prognostic insight. Furthermore, although combined models showed statistically significant differences in AUROC, the absolute improvement in discrimination was modest and may not translate into clinically meaningful benefit at the individual patient level. Accordingly, the term “improved discrimination” should be interpreted in a statistical rather than a definitive clinical sense. Finally, external validation was not performed, and the predominantly older study population may limit the applicability of these findings to younger ICU patients; prospective multicenter studies are needed to clarify the clinical impact of implementing these indices in routine ICU practice.

## 5. Conclusions

In critically ill patients admitted to the intensive care unit with pneumonia, mortality risk is not adequately explained by physiological severity alone. The integration of NEWS with indices reflecting nutritional–immune reserve (PNI) or endothelial stress (EASIX) provides superior prognostic discrimination compared with single-score approaches. These combined models rely on routinely available clinical and laboratory parameters and are readily applicable at the bedside. A multidimensional approach to early risk stratification may facilitate earlier identification of high-risk patients and support more individualized management strategies. Further prospective and multicenter studies are warranted to validate these findings and assess their impact on clinical outcomes.

## Figures and Tables

**Table 1:** Baseline characteristics of intensive care unit patients with pneumonia according to in-hospital mortality.

Variables	All Patients (n = 221)	Survivors (n = 79)	Non-Survivors (n = 142)	*p*
Age, years	74.8 ± 14.1	73.1 ± 16.3	75.7 ± 12.7	0.193
Male sex, n (%)	119 (53.8)	39 (49.4)	80 (56.3)	0.319
Nursing home resident, n (%)	7 (3.2)	2 (2.5)	5 (3.5)	0.514
Comorbidity, n (%)	208 (94.1)	71 (89.9)	137 (96.5)	0.047
Diabetes mellitus, n (%)	58 (26.2)	22 (27.8)	36 (25.4)	0.686
COPD, n (%)	34 (15.4)	12 (15.2)	22 (15.5)	0.952
Malignancy, n (%)	63 (28.5)	23 (29.1)	40 (28.2)	0.881
Immunosuppression, n (%)	37 (16.7)	10 (12.7)	27 (19.0)	0.225
Chronic kidney disease, n (%)	28 (12.7)	12 (15.2)	16 (11.3)	0.401
Chronic liver disease, n (%)	2 (0.9)	1 (1.3)	1 (0.7)	0.673
Heart failure, n (%)	24 (10.9)	9 (11.4)	15 (10.6)	0.507
Stroke, n (%)	40 (18.1)	32 (22.5)	8 (10.1)	0.022
Coronary artery disease, n (%)	37 (16.7)	14 (17.7)	23 (16.2)	0.771
Psychosis, n (%)	2 (0.9)	1 (1.3)	1 (0.7)	0.673
Other, n (%)	157 (71.0)	51 (64.6)	106 (74.6)	0.113
PNI	33.9 (29.9–37.9)	35.3 (31.5–39.6)	31.5 (28.4–37.4)	0.001
EASIX	2.2 (1.1–4.9)	2.0 (0.8–3.6)	2.6 (1.2–6.8)	0.021
NEWS	9.0 ± 3.1	7.8 ± 3.1	9.6 ± 2.9	<0.001
Length of ICU stay, days	8.0 (5.0–16.0)	8.0 (4.5–10.5)	9.0 (5.0–21.0)	0.055
Intubation, n (%)	136 (61.5)	19 (24.1)	117 (82.4)	<0.001
Inotrope use, n (%)	98 (44.3)	14 (17.7)	84 (59.2)	<0.001
pH	7.38 (7.28–7.44)	7.40 (7.32–7.45)	7.37 (7.26–7.43)	0.016
Lactate, mmol/L	1.8 (1.3–3.0)	1.4 (1.0–1.9)	2.3 (1.5–3.7)	<0.001
Urea, mg/dL	88.0 (58.0–140.0)	76.0 (56.5–128.0)	96.0 (63.0–143.0)	0.056
WBC, ×10^3^/µL	12.4 (8.9–17.7)	11.0 (8.8–16.0)	13.8 (8.9–18.6)	0.062
Albumin, g/dL	2.9 (2.5–3.3)	3.1 (2.8–3.4)	2.8 (2.4–3.1)	<0.001
Procalcitonin, ng/mL	1.9 (0.6–9.0)	0.9 (0.3–2.7)	3.6 (1.1–12.9)	<0.001
CRP, mg/L	155.0 (83.0–240.0)	122.0 (65.7–250.0)	167.5 (97.0–240.0)	0.088
Ferritin, µg/L	381.0 (153.0–933.0)	279.0 (100.0–500.0)	451.0 (189.0–1035.5)	0.007
D-dimer, mg/L	3.0 (1.6–6.8)	2.9 (1.5–4.7)	3.3 (1.6–8.1)	0.090

Data are presented as mean ± standard deviation or median (interquartile range) for continuous variables and as number (percentage) for categorical variables. Comparisons between survivors and non-survivors were performed using Student’s *t*-test or Mann–Whitney *U* test for continuous variables and the chi-square test or Fisher’s exact test for categorical variables, as appropriate. Abbreviations: PNI: Prognostic Nutritional Index; EASIX: Endothelial Activation and Stress Index; NEWS: National Early Warning Score; ICU: intensive care unit; COPD: chronic obstructive pulmonary disease; WBC: white blood cell count; CRP: C-reactive protein. A *p* value < 0.05 was considered statistically significant.

**Table 2:** Univariate and multivariate logistic regression analyses of baseline and clinical course-related variables associated with in-hospital mortality.

	In-Hospital Mortality (n = 142)							
	Univariate Analysis				Multivariate Analysis			
Variable	OR (95% CI)	Wald	β	*p*-Value	OR (95% CI)	Wald	β	*p*-Value
Age	1.013 (0.993–1.033)	1.685	0.013	0.194				
Sex (female, ref.)	0.756 (0.435–1.312)	0.991	−0.280	0.320				
Comorbidity	3.087 (0.974–9.785)	3.669	1.127	0.055				
PNI	0.968 (0.937–0.999)	3.964	−0.033	0.046				
EASIX	1.093 (1.019–1.171)	6.248	0.089	0.012				
NEWS	1.215 (1.101–1.340)	15.157	0.194	<0.001				
Length of ICU stay	1.040 (1.007–1.073)	5.895	0.039	0.015				
Intubation	14.779 (7.541–28.963)	61.550	2.693	<0.001	12.460 (4.662–33.303)	25.288	2.522	<0.001
Inotrope use	6.724 (3.450–13.107)	31.319	1.906	<0.001	5.139 (1.996–13.233)	11.505	1.637	0.001
Lactate	1.788 (1.356–2.357)	16.952	0.581	<0.001	1.746 (1.214–2.512)	9.046	0.558	0.003
Urea	1.003 (0.999–1.008)	2.164	0.003	0.141				
WBC	1.027 (0.993–1.062)	2.391	0.026	0.122				
Albumin	0.321 (0.188–0.549)	17.264	−1.136	<0.001				
Procalcitonin	1.016 (1.001–1.031)	4.662	0.016	0.031				
CRP	1.002 (0.999–1.005)	2.366	0.002	0.124				
Ferritin	1.001 (1.000–1.001)	5.567	0.001	0.018				
D-dimer	1.021 (0.984–1.060)	1.275	0.021	0.259				

Univariate and multivariate logistic regression analyses were performed to identify factors associated with in-hospital mortality. Variables with a *p* value < 0.10 in univariate analysis were entered into the multivariate model. Results are presented as odds ratios (ORs) with 95% confidence intervals (CIs). The Wald statistic, regression coefficient (B-value), and corresponding *p* values are shown. Abbreviations: PNI: Prognostic Nutritional Index; EASIX: Endothelial Activation and Stress Index; NEWS: National Early Warning Score; ICU: intensive care unit; WBC: white blood cell count; CRP: C-reactive protein. A two-sided *p* value < 0.05 was considered statistically significant.

**Table 3:** Interaction analysis of NEWS with PNI and EASIX in predicting in-hospital mortality among intensive care unit patients with pneumonia.

		Odds Ratio (95% CI)			
	n Died/n Total (%)	Crude Model	*p*	Adjusted Model	*p*
NEWS/PNI					
NEWS ≤ 7/PNI ≥ 34.7	10/27 (37.0)	Reference	-	Reference	-
NEWS > 7/PNI ≥ 34.7	43/74 (58.1)	2.358 (0.952–5.843)	0.064	2.533 (1.011–6.349)	0.047
NEWS ≤ 7/PNI < 34.7	12/22 (54.5)	2.040 (0.648–6.420)	0.223	2.170 (0.681–6.912)	0.190
NEWS > 7/PNI < 34.7	77/98 (78.6)	6.233 (2.489–15.612)	<0.001	6.445 (2.551–16.283)	<0.001
NEWS/EASIX					
NEWS ≤ 7/EASIX < 3.3	11/30 (36.7)	Reference	-	Reference	-
NEWS > 7/EASIX < 3.3	66/104 (63.5)	3.000 (1.291–6.970)	0.011	3.152 (1.340–7.417)	0.009
NEWS ≤ 7/EASIX ≥ 3.3	11/19 (57.9)	2.375 (0.733–7.691)	0.149	2.515 (0.766–8.261)	0.128
NEWS > 7/EASIX ≥ 3.3	54/68 (79.4)	6.662 (2.584–17.176)	<0.001	7.365 (2.773–19.558)	<0.001

Abbreviations: ICU, Intensive Care Unit; NEWS, National Early Warning Score; PNI, Prognostic Nutritional Index; EASIX, Endothelial Activation and Stress Index; OR, Odds Ratio; CI, Confidence Interval; WBC, White Blood Cell count; CRP, C-reactive Protein; COPD, Chronic Obstructive Pulmonary Disease.

**Table 4:** Discriminative performance of NEWS, PNI, EASIX, and their combined models for predicting in-hospital mortality in intensive care unit patients with pneumonia.

Prognostic Models			Pairwise Analysis				
			DBA	95% CI	SE	Z Statistic	*p* Value
	Without PNI, AUROC 95% CI	With PNI, AUROC 95% CI					
Base Model	0.737 (0.671–0.803)	0.760 (0.697–0.823)	−0.023	(−0.050–0.004)	0.253	−1.657	0.093
NEWS	0.657 (0.581–0.732)	0.690 (0.617–0.763)	−0.033	(−0.072–0.005)	0.271	−1.697	0.090
Base Model + NEWS	0.763 (0.699–0.827)	0.791 (0.731–0.852)	−0.028	(−0.061–0.005)	0.249	−1.647	0.099
	Without EASIX, AUROC 95% CI	With EASIX, AUROC 95% CI					
Base Model	0.737 (0.671–0.803)	0.752 (0.688–0.816)	−0.015	(−0.031–0.001)	0.254	−1.878	0.060
NEWS	0.657 (0.581–0.732)	0.709 (0.640–0.779)	−0.053	(−0.092–0.013)	0.268	−2.606	0.009
Base Model + NEWS	0.763 (0.699–0.827)	0.785 (0.724–0.845)	−0.022	(−0.045–0.002)	0.249	−1.824	0.068

Abbreviations: AUROC, Area Under the Receiver Operating Characteristic curve; CI, Confidence Interval; DBA, Difference Between AUROCs; SE, Standard Error. Base Model = Age, Sex, Lactate, Procalcitonin, and CRP.

**Table 5:** Incremental discriminative value of adding PNI or EASIX to a NEWS-based base model for predicting in-hospital mortality in intensive care unit patients with pneumonia.

Prognostic Model		Pairwise Analysis				
	AUROC 95% CI	DBA	95% CI	SE	Z Statistic	*p* Value
Base Model vs. Base Model + NEWS + PNI	0.737 (0.671–0.803) vs. 0.791 (0.731–0.852)	−0.054	(−0.102, −0.007)	0.252	−2.249	0.025
Base Model vs. Base Model + NEWS + EASIX	0.737 (0.671–0.803) vs. 0.785 (0.724–0.845)	−0.048	(−0.090, −0.005)	0.251	−2.211	0.027

Abbreviations: AUROC, Area Under the Receiver Operating Characteristic curve; CI, Confidence Interval; DBA, Difference Between AUROCs; SE, Standard Error; NEWS, National Early Warning Score; PNI, Prognostic Nutritional Index; EASIX, Endothelial Activation and Stress Index.

## Data Availability

Data are available from the corresponding author upon reasonable request.

## Supplementary Materials

The following supporting information can be downloaded at: https://www.mdpi.com/article/10.3390/medicina62010207/s1, Table S1: Exclusion criteria and number of patients excluded at each step during ICU cohort selection.

## Abbreviations

The following abbreviations are used in this manuscript:

**Abbreviation**	**Definition**
ICU	Intensive Care Unit
NEWS	National Early Warning Score
PNI	Prognostic Nutritional Index
EASIX	Endothelial Activation and Stress Index
AUROC	Area Under the Receiver Operating Characteristic Curve
CI	Confidence Interval
OR	Odds Ratio
CRP	C-reactive Protein
WBC	White Blood Cell
COPD	Chronic Obstructive Pulmonary Disease
