# Supplementary material for: Prognostic Value of the National Early Warning Score Combined with Nutritional and Endothelial Stress Indices for Mortality Prediction in Critically Ill Patients with Pneumonia
Source: Medicina (Kaunas). 2026 Jan 19;62(1):207. doi: 10.3390/medicina62010207 (PMC12843635; doi:10.3390/medicina62010207)
Supplement: Supplementary file 1 [file medicina-62-00207-s001.zip › medicina-4093318-supplementary.pdf]

Table S1. Exclusion criteria and number of patients excluded at each step during ICU cohort selection.

Exclusion Criterion	Number of Patients (n)
Age <18 years	0
ICU admission for reasons other than pneumonia (primary diagnosis not pneumonia)	547
Transferred/discharged to another center within the first 24 h of ICU admission	2
Missing data required for NEWS calculation	0
Missing albumin and/or lymphocyte count required for PNI calculation	0
Missing LDH and/or creatinine and/or platelet count required for EASIX calculation	0
Duplicate ICU admissions/readmissions (only first ICU admission included)	14

Exclusion criteria were applied sequentially during patient screening and eligibility assessment.
